# USP25 Inhibits Neuroinflammatory Responses After Cerebral Ischemic Stroke by Deubiquitinating TAB2

**DOI:** 10.1002/advs.202301641

**Published:** 2023-08-16

**Authors:** Zhongding Li, Baohua Liu, Kate Lykke Lambertsen, Bettina Hjelm Clausen, Zhenhu Zhu, Xue Du, Yanqi Xu, Frantz Rom Poulsen, Bo Halle, Christian Bonde, Meng Chen, Xue Wang, Dirk Schlüter, Jingyong Huang, Ari Waisman, Weihong Song, Xu Wang

**Affiliations:** ^1^ Oujiang Laboratory (Zhejiang Lab for Regenerative Medicine, Vision and Brain Health) School of Pharmaceutical Sciences Wenzhou Medical University Wenzhou 325035 China; ^2^ Department of Neurological Rehabilitation The Second Affiliated Hospital and Yuying Children's Hospital of Wenzhou Medical University Wenzhou 325027 China; ^3^ Department of Neurobiology Research Institute of Molecular Medicine University of Southern Denmark Odense C 5000 Denmark; ^4^ BRIDGE – Brain Research – Inter Disciplinary Guided Excellence Department of Clinical Research University of Southern Denmark Odense C 5000 Denmark; ^5^ Department of Neurology Odense University Hospital Odense C 5000 Denmark; ^6^ School of Pharmaceutical Sciences Wenzhou Medical University Wenzhou 325035 China; ^7^ Department of Neurosurgery Odense University Hospital Odense C 5000 Denmark; ^8^ Institute of Medical Microbiology and Hospital Epidemiology Hannover Medical School 30625 Hannover Germany; ^9^ Department of Vascular Surgery The First Affiliated Hospital of Wenzhou Medical University Wenzhou 325015 China; ^10^ Institute for Molecular Medicine Johannes Gutenberg University Mainz 55131 Mainz Germany; ^11^ Key Laboratory of Alzheimer's Disease of Zhejiang Province Institute of Aging Wenzhou Medical University Wenzhou 325035 China

**Keywords:** cerebral ischemic stroke, microglia, neuroinflammation, ubiquitination, USP25

## Abstract

Cerebral ischemic stroke is a leading cause of morbidity and mortality globally. However, the mechanisms underlying ischemic stroke injury remain poorly understood. Here, it is found that deficiency of the ubiquitin‐specific protease USP25 significantly aggravate ischemic stroke injury in mice. USP25 has no impact on neuronal death under hypoxic conditions, but reduced ischemic stroke‐induced neuronal loss and neurological deficits by inhibiting microglia‐mediated neuroinflammation. Mechanistically, USP25 restricts the activation of NF‐κB and MAPK signaling by regulating TAB2. As a deubiquitinating enzyme, USP25 removeds K63‐specific polyubiquitin chains from TAB2. AAV9‐mediated TAB2 knockdown ameliorates ischemic stroke injury and abolishes the effect of USP25 deletion. In both mouse and human brains, USP25 is markedly upregulated in microglia in the ischemic penumbra, implying a clinical relevance of USP25 in ischemic stroke. Collectively, USP25 is identified as a critical inhibitor of ischemic stroke injury and this data suggest USP25 may serve as a therapeutic target for ischemic stroke.

## Graphical abstract

In this study, the authors identify USP25, a deubiquitinating enzyme, as a key inhibitor of cerebral ischemic stroke injury. USP25 is upregulated in microglia in both humans and mice after ischemic stroke. In mice, knockout of USP25 increases microglia‐mediated neuroinflammation and thereby significantly exacerbates stroke‐induced neurological deficit. Therefore, USP25 may serve as a potential therapeutic target for ischemic stroke.

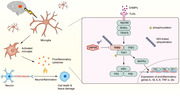

## 1 Introduction

Cerebral stroke is a life‐threatening cerebrovascular disease associated with high morbidity, disability, and mortality. Cerebral stroke can be divided into ischemic stroke and hemorrhagic stroke, with ischemic stroke accounting for > 80% of total stroke cases.^[^ ^1^ ^]^ Current therapeutic approaches to restore blood supply in ischemic stroke are limited to intravenous thrombolysis with tissue plasminogen activator (tPA) and mechanical thrombectomy.^[^ ^2^ ^]^ However, reperfusion of the ischemic brain can cause a detrimental secondary brain injury, called cerebral ischemia‐reperfusion injury, leading to further tissue damage and dysfunction.^[^ ^3^ ^]^ Unraveling the mechanism and identification of novel factors underlying the pathophysiology of stroke‐induced injury is beneficial for the development of efficacious therapies for this life‐threatening disease.

Multiple mechanisms, such as excitotoxicity, calcium overload, oxidative stress, and neuroinflammation, synergistically contribute to the pathogenesis of ischemic stroke injury.^[^ ^4^ ^]^ Of note, inflammation is a key factor associated with secondary injury in ischemic stroke.^[^ ^5^ ^]^ After ischemic stroke, damage‐associated molecular patterns (DAMPs), for example HMGB‐1 and S100A8/A9, are released from damaged and necroptotic cells in the primary attack.^[^ ^6^ ^]^ These DAMPs are sensed by adjacent brain‐resident cells, particularly microglia, through pattern recognition receptors (PRRs).^[^ ^7^ ^]^ The interplay between DAMPs and microglial PRRs activates intracellular proinflammatory signaling pathways, such as the NF‐κB pathway, resulting in the production of proinflammatory cytokines and chemokines.

The activation of cell signaling is tightly controlled by post‐translational modifications (PTMs). Ubiquitination is an important PTM that regulates a broad range of biological activities, especially inflammatory responses.^[^ ^8^ ^]^ Ubiquitination is catalyzed sequentially by ubiquitin‐activating enzymes (E1s), ubiquitin‐conjugating enzymes (E2s), and ubiquitin ligases (E3s), and reversed by deubiquitinating enzymes (DUBs).^[^ ^9^ ^]^ In recent years, DUBs have emerged as key regulators of neuroinflammation.^[^ ^10^ ^]^ USP25 is a DUB that is closely related to inflammatory responses. For example, USP25 inhibits experimental autoimmune encephalomyelitis by inhibiting IL‐17‐induced signaling and inflammation.^[^ ^11^ ^]^ In addition, USP25 is also involved in other inflammation‐associated diseases including sepsis,^[^ ^12^ ^]^ viral infection,^[^ ^13^ ^]^ pancreatitis,^[^ ^14^ ^]^ colitis,^[^ ^15^ ^]^ and Alzheimer's disease.^[^ ^16^ ^]^ However, the function of USP25 in stroke has not been studied.

In this study, we found that USP25 deficiency significantly increased infarct size and exacerbated neurological deficits induced by transient middle cerebral artery occlusion (MCAO) in mice. USP25 inhibited MCAO‐induced neuroinflammation by inhibiting the inflammatory activation of microglia. Mechanistically, USP25 inhibited NF‐κB activation by reducing the K63 polyubiquitination of TAB2. Moreover, a compensatory upregulation of USP25 was observed in microglia after ischemic stroke in both mice and humans. Thus, USP25 serves as a new and potent inhibitor of neuroinflammation after ischemic stroke, providing a novel therapeutic target for treating ischemic stroke.

## 2 Results

### 2.1 USP25 Ameliorates Ischemic Stroke Injury in Mice

**Figure 1:**
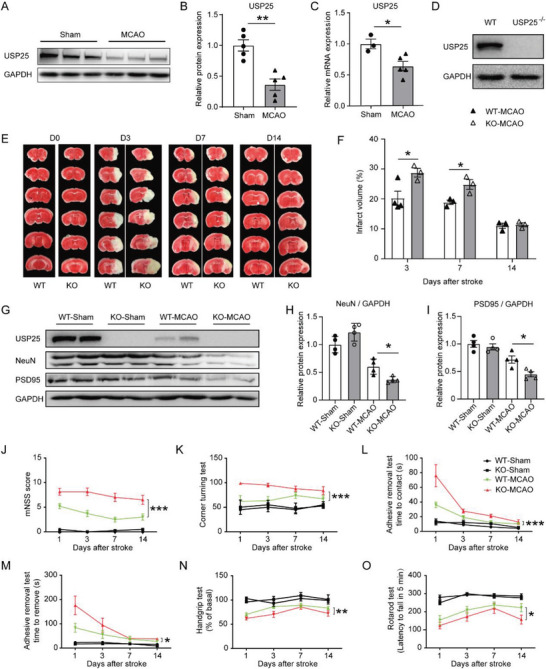
USP25 deficiency aggravates cerebral damage and neurological deficits in MCAO mice. A,B) Representative western blot images A) and quantification B) of USP25 protein abundance in the ischemic brain hemisphere on day 3 after MCAO. *n* = 5 mice per group. C) Relative transcription levels of USP25 in the ischemic brain hemisphere on day 2 after MCAO was determined by qRT‐PCR. *n* = 3 to 5 mice per group. D) Western blot analysis of USP25 expression in the brain of WT and USP25^−/−^ mice. E,F) Representative TTC staining E) and percentages of infarct volume F) on day 0, 3, 7, 14 after MCAO. *n* = 3 to 4 mice per group. G–I) The protein levels of NeuN and PSD95 in the ischemic brain hemisphere on day 2 after MCAO were analyzed by western blot G). The relative protein levels of H) NeuN and I) PSD95 were calculated after normalization to GAPDH. *n* = 4 mice per group. J–O) Neurobehavioral functions were assessed with J) mNSS test, K) corner turning test, L,M) adhesive removal test, N) handgrip test, and O) rotarod test on day 1, 3, 7, 14 after MCAO. *n* = 4 to 10 mice per group. Data are displayed as the mean ± SEM.* *P* < 0.05; **P < 0.01; ***P < 0.001.

Given that USP25 is a DUB that participates in the regulation of various inflammatory diseases^[^ ^11^, ^12^, ^13^, ^14^, ^15^ ^]^ and that neuroinflammation is also a critical factor underlying ischemic stroke injury, we asked whether USP25 was involved in ischemic stroke injury. We first analyzed the expression of USP25 in mouse brains before and after MCAO and found that both protein and mRNA levels of USP25 were significantly reduced in the brain of MCAO mice (**Figure** **1**A–C), implying a potential involvement of USP25 in ischemic stroke. To confirm the role of USP25 in ischemic stroke, transient MCAO was induced in wild type C57BL/6 (WT) mice and USP25 knockout (USP25^−/−^) mice (Figure 1D). Ablation of USP25 significantly enlarged the infarct volume on day 3 and 7 after reperfusion (Figure 1E,F), suggesting that USP25 alleviates cerebral injury induced by ischemic stroke. Consistently, we found that USP25 deficiency aggravated MCAO‐induced neuronal damage, as protein levels of NeuN and PSD95 were significantly lower in infarcted brains of USP25^−/‐^ mice as compared with that in WT mice after ischemic stroke (Figure 1G–I). In addition, behavioral assessments, including mNSS test, corner turning test, adhesive removal test, handgrip test, and rotarod test, showed that USP25^−/‐^ mice had more severe neurological deficits than WT mice after MCAO (Figure 1J–O). Collectively, these results show that USP25 deficiency increases MCAO‐induced infarct volume, neuronal damage, and neurological deficits in mice, indicating a protective role of USP25 in ischemic stroke injury.

### 2.2 USP25 Inhibits Inflammatory Activation of Microglia after Ischemic Stroke

**Figure 2:**
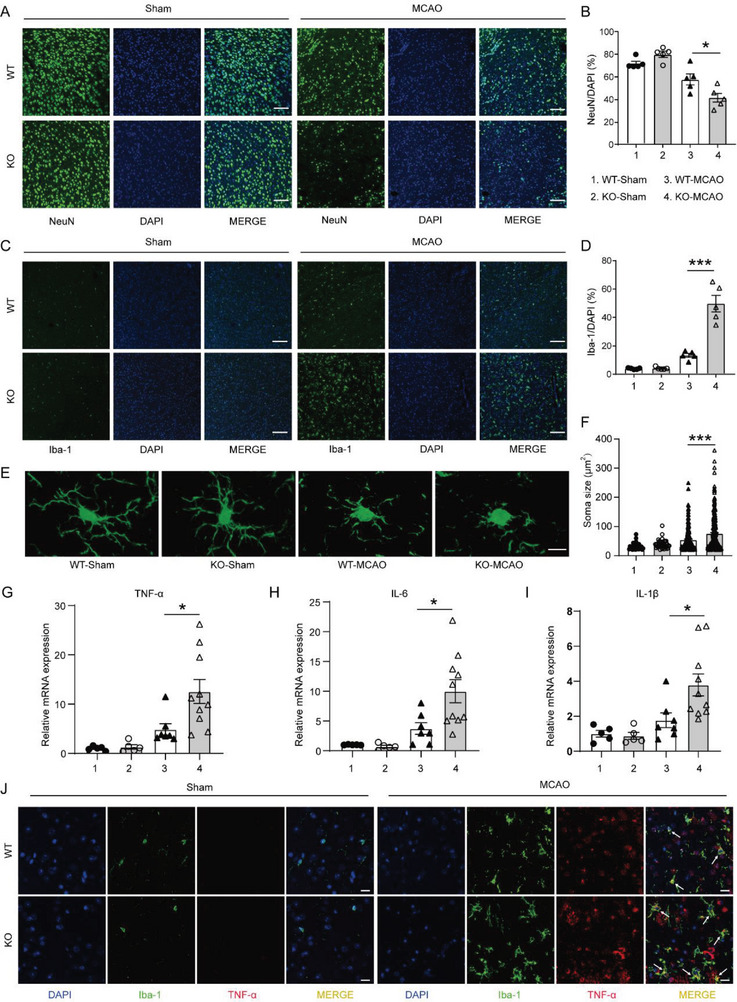
USP25 deficiency enhances microglial activation and neuroinflammation in MCAO mice. A,B) Representative immunofluorescence staining A) and percentages B) of NeuN^+^ cells in the ischemic penumbra on day 2 after MCAO. Scale bar, 100 µm, *n* = 5 mice per group. C,D) Representative immunofluorescence staining C) and percentages D) of Iba‐1^+^ cells in the ischemic penumbra on day 2 after MCAO. Scale bar, 100 µm. *n* = 5 mice per group. E,F) Representative Z‐stack confocal images E) and quantification of soma size F) of Iba1^+^ microglia in the ischemic penumbra on day 2 after MCAO. Scale bar, 10 µm. *n* = 29 to 264 microglia per group were counted. G–I) qRT‐PCR analysis of TNF‐α G), IL‐6 H), and IL‐1β I) transcription in the ischemic brain hemisphere on day 2 after MCAO. *n* = 5 to 10 mice per group. J) Representative immunofluorescence staining of Iba‐1 (green) and TNF‐α (red) in the ischemic penumbra on day 2 after MCAO. Scale bar, 20 µm. Data represent the mean ± SEM. * *P* < 0.05; **P < 0.01; ***P < 0.001.

Since neuronal death is the core event in ischemic stroke injury, we analyzed the influence of MCAO on neurons with immunofluorescence (**Figure** **2**A,B). The number of neurons was reduced after MCAO in the ischemic penumbra, as compared to sham, and USP25 deficiency further exacerbated the loss of neurons after ischemia (Figure 2B), which is in line with the finding that USP25^−/−^ mice displayed more severe neuronal damage than WT mice after MCAO (Figure 1G–I). Double immunofluorescence staining demonstrated that USP25 was mainly expressed in neurons in the healthy mouse brain (Figure S1A, Supporting Information). It is possible that USP25 may directly regulate neuronal death after stroke. However, both knockdown and pharmacological inhibition of USP25 had no impact on the death of SH‐SY5Y cells induced by OGD (Figure S1B–H, Supporting Information). Similarly, USP25 did not affect OGD‐induced production of DAMPs such as S100A8/A9 in neurons (Figure S1I,J, Supporting Information). These findings show that USP25 ameliorates ischemic stroke injury not by directly regulating neurons.

Ischemic stroke induces the migration, proliferation, and activation of microglia, which are brain‐resident immune cells, in the infarcted area and activated microglia contribute to the establishment of an inflammatory milieu that is detrimental to neurons.^[^ ^17^ ^]^ We found that, after MCAO, USP25^−/−^ mice had significantly more microglia in the infarcted penumbra than WT mice (Figure 2C,D). Of note, deletion of USP25 had no influence on the survival of microglia upon OGD treatment (Figure S2, Supporting Information), indicating that the increased numbers of microglia in USP25^−/−^ mice were not caused by reduced microglia death after MCAO. Astrocytes are another group of glial cells with inflammatory potential in the brain and deletion of USP25 also increased the number of astrocytes in the infarcted penumbra (Figure S3A,B, Supporting Information). In addition to cell number, microglia undergo morphological changes, such as soma enlargement and process shortening, upon activation.^[^ ^18^ ^]^ Using 3D reconstruction, we found that MCAO induced the enlargement of microglia soma, which was further increased by the ablation of USP25 (Figure 2E,F), suggesting that USP25 deficiency promotes the activation of microglia after ischemic attack. Activated microglia are the primary source of TNF‐α, IL‐6, and IL‐1β in the brain, and the mRNA levels of all of these cytokines were significantly elevated in the infarcted brain hemisphere of USP25^−/−^ mice (Figure 2G–I), showing that USP25 alleviates MCAO‐induced microglia‐mediated neuroinflammation. In addition, double immunofluorescence staining confirmed that USP25‐deficient microglia produced more TNF‐α than USP25‐sufficient microglia in vivo after transient MCAO (Figure 2J). In aggregate, these results show that USP25 inhibits MCAO‐induced glial cell activation and neuroinflammation.

### 2.3 USP25 Inhibits Cerebral Infiltration of Immune Cells after Ischemic Stroke

**Figure 3:**
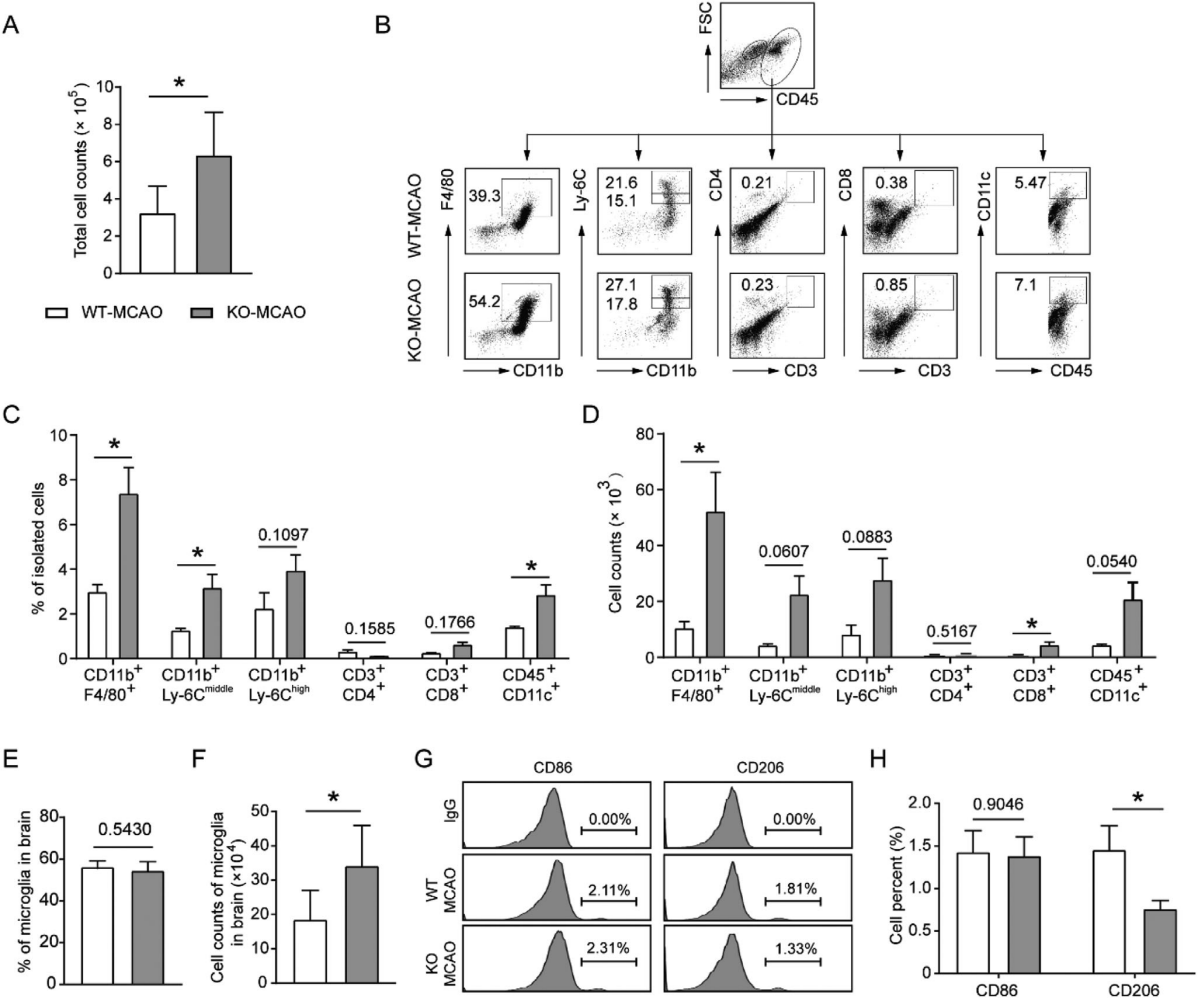
USP25 deficiency increases the infiltration of immune cells in the ischemic brain of MCAO mice. A) The absolute number of CD45^+^ cells in the ischemic brain hemisphere of WT and USP25^−/‐^ mice on day 2 after MCAO. B) Gating strategy and representative dot plots of different cell populations. C,D) Percentages C) and absolute numbers D) of infiltrating leukocytes. E,F) Percentages E) and absolute numbers F) of microglia. G,H) Representative histogram G) and percentages H) of CD86^+^ and CD206^+^ microglia. Data represent the mean ± SEM. *n* = 5 to 7 mice per group. * *P* < 0.05; The *P* values > 0.05 were given in numbers.

After ischemic stroke, damaged tissue and neuroinflammation facilitates the recruitment of peripheral immune cells, further amplifying inflammatory responses in the brain.^[^ ^19^ ^]^ Therefore, we isolated and analyzed immune cells in infarcted brain hemispheres. As compared with WT MCAO mice, more immune cells were detected in USP25^−/−^ MCAO mice (**Figure** **3**A). Flow cytometry was applied to dissect the composition of cells (Figure 3B–H). Higher percentages and absolute numbers of infiltrating macrophages, which are key players in ischemic stroke, were detected in injured hemispheres of USP25^−/−^ mice (Figure 3B–D). Although the percentage of microglia did not differ between the two genotypes, the absolute number of microglia was significantly higher in USP25^−/−^ mice due to the increased number of total cells (Figure 3A,E,F), supporting the finding that USP25^−/−^ mice had more microglia in the infarcted penumbra as shown in Figure 2C,D. Microglia are generally classified into M1 and M2 phenotypes. The M1 microglia (CD86^+^) are proinflammatory whereas the M2 microglia (CD206^+^) play an anti‐inflammatory role. USP25 deficiency significantly impaired the M2 differentiation of microglia (Figure 3G,H), consolidating an anti‐inflammatory function of USP25 in microglia. Taken together, these findings demonstrate that USP25 alleviates MCAO‐induced neuroinflammation by regulating both microglia and infiltrating peripheral immune cells.

### 2.4 USP25 is Upregulated in Microglia in Response to Ischemia

**Figure 4:**
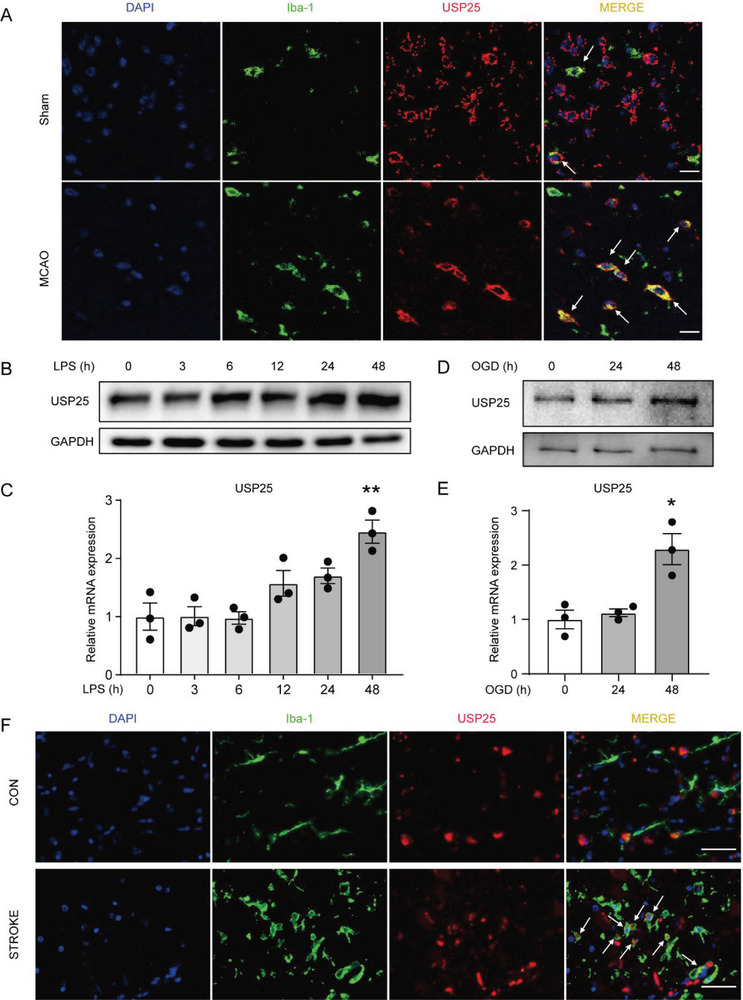
Upregulation of USP25 in microglia after ischemic stroke. A) Representative immunostaining of USP25 (red) and Iba‐1 (green) in the ischemic penumbra on day 2 after MCAO. Scale bar, 10 µm. B,C) Western blot B) and qRT‐PCR C) was performed to detect USP25 expression in BV2 cell after stimulation with LPS for the indicated time periods. *n* = 3 per group. D,E) Western blot D) and qRT‐PCR E) was performed to detect USP25 expression in BV2 cell after OGD for the indicated time periods. *n* = 3 per group. F) Representative immunostaining of USP25 (red) and Iba‐1 (green) in the normal temporal lobe brain tissue from a 78‐year‐old man and brain tissue from a 67‐year‐old man with a >8 day ischemic stroke in the temporal lobe. Scale bar, 40 µm. Data are shown as the mean ± SEM. * *P* < 0.05; **P < 0.01.

In homeostatic brains, USP25 was mainly expressed in neurons (Figure S1A, Supporting Information) and only a few microglia expressed low levels of USP25 (**Figure** **4**A). However, USP25 was strongly upregulated in microglia after MCAO (Figure 4A). After ischemic stroke, microglia recognize DAMPs released from damaged or dead cells mainly through toll‐like receptors (TLRs), leading to the production of inflammatory mediators.

Consistent with the in vivo finding, both protein and mRNA levels of USP25 were upregulated in BV2 cells upon stimulation with LPS, a strong TLR4 agonist (Figure 4B,C). In addition to LPS, OGD could also upregulate USP25 in BV2 cells in vitro (Figure 4D,E), further confirming our in vivo findings. Interestingly, USP25 expression was markedly upregulated in microglia in the brains of patients with ischemic stroke (Figure 4F). Together, these results show that USP25 is upregulated in microglia in response to the stress of ischemic stroke, probably serving as a negative‐feedback mechanism to inhibit the activation of microglia.

### 2.5 USP25 Inhibits TLR4‐Induced Cytokine Production in Microglia

**Figure 5:**
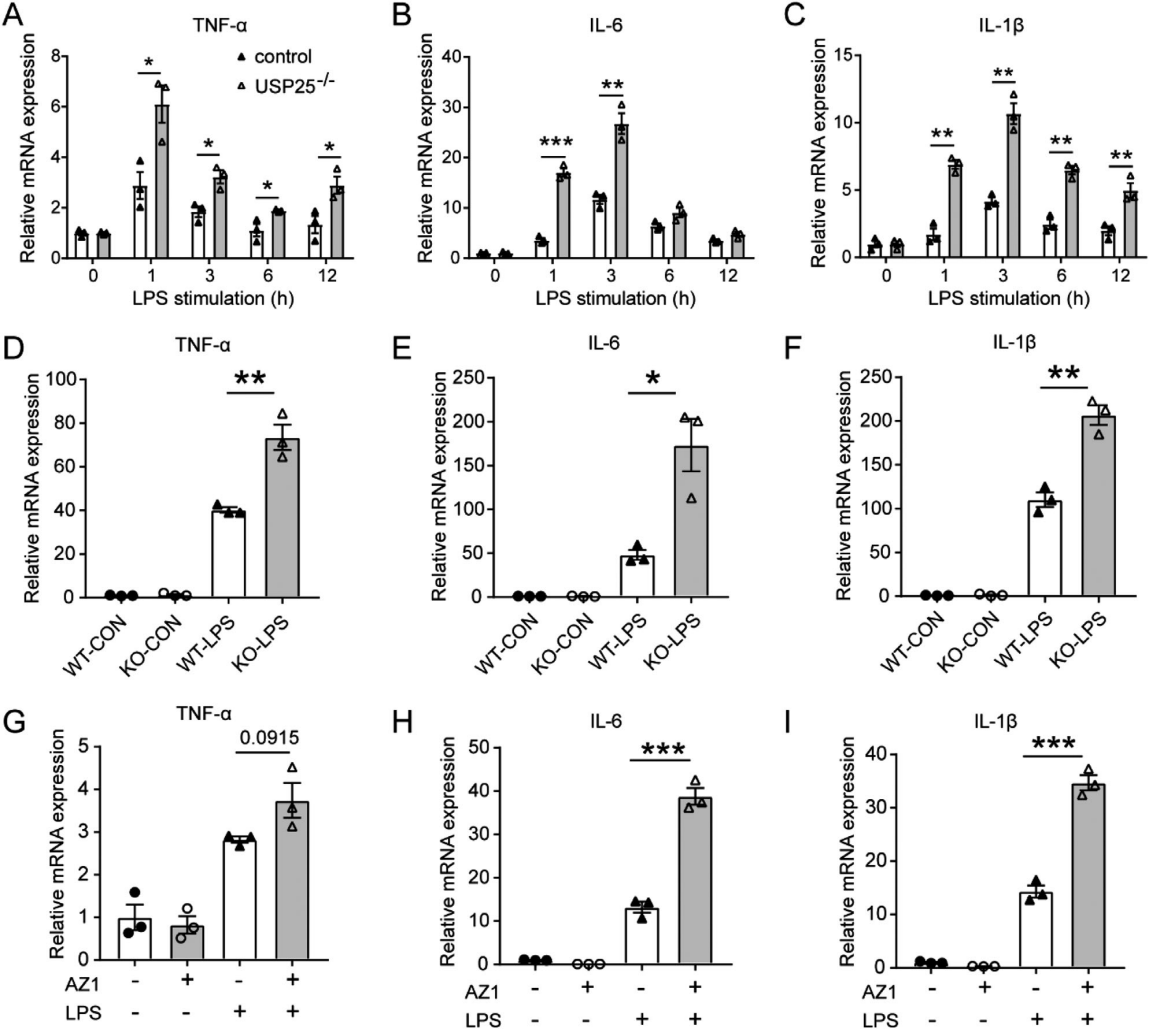
USP25 deficiency enhances LPS‐induced cytokine production in microglia. A–C) Transcription of A) TNF‐α, B) IL‐6, and C) IL‐1β in control and USP25^−/−^ BV2 cells was analyzed by qRT‐PCR after stimulation with LPS (500 ng ml^−1^) for the indicated time periods. D–F) Transcription of D) TNF‐α, E) IL‐6, and F) IL‐1β in control and USP25^−/−^ primary microglia was detected by qRT‐PCR after stimulation with LPS (500 ng ml^−1^) for 3 h. G–I) BV2 cells were pretreated with AZ1 (5 µm) for 2 h, followed by stimulation with LPS (500 ng ml^−1^) for 3 h. The transcription of G) TNF‐α, H) IL‐6, and I) IL‐1β was detected by qRT‐PCR. All data represent the mean ± SEM. *n* = 3 per group. * *P* < 0.05; **P < 0.01; ***P < 0.001.

To evaluate the effect of USP25 in microglia during inflammatory responses, we generated a USP25^−/−^ BV2 cell line using the CRISPR/Cas9 technology (Figure S4, Supporting Information). Upon stimulation with LPS, USP25^−/−^ BV2 cells produced significantly more TNF‐α, IL‐6, and IL‐1β than USP25‐sufficient control cells (**Figure** **5**A–C). To confirm these findings, we isolated primary microglia from WT and USP25^−/−^ mice and stimulated them with LPS. As compared with WT microglia, USP25^−/−^ primary microglia produced significantly more TNF‐α, IL‐6, and IL‐1β in response to LPS (Figure 5D–F), in line with the results obtained with BV2 cells. Similarly, USP25 deficiency enhanced cytokine production in primary astrocytes upon LPS stimulation (Figure S3, Supporting Information), indicating that USP25 plays a general anti‐inflammatory role in glial cells. In addition, pharmacological inhibition of USP25 with the small‐molecule inhibitor AZ1 also enhanced the production of cytokines and chemokines in BV2 cells (Figure 5G–I; Figure S5, Supporting Information). These in vitro data are in accordance with the in vivo data that microglia activation and neuroinflammation was increased in the infarcted brain hemisphere of USP25^−/−^ mice (Figure 2), showing that USP25 inhibits the inflammatory activation of microglia.

### 2.6 USP25 Negatively Regulates Inflammatory Signal Transduction in Microglia

**Figure 6:**
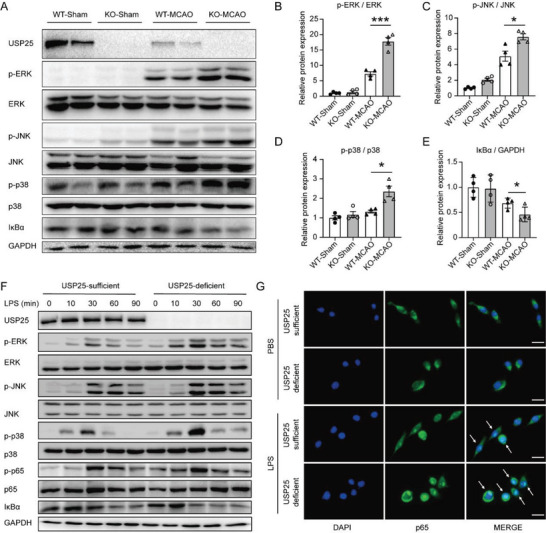
Ablation of USP25 promotes NF‐κB and MAPK activation. A–E) Representative immunoblots A) and quantification B–E) of indicated signaling molecules in the ischemic brain hemisphere on day 2 after MCAO. *n* = 4 mice per group. F) Representative immunoblots of indicated signaling molecules in USP25‐sufficient and ‐deficient BV2 cells treated with LPS (500 ng ml^−1^) for 0, 10, 30, 60, and 90 min. G) Representative immunostaining of p65 (green) in USP25‐sufficient and ‐deficient BV2 cells treated with LPS (500 ng ml^−1^) for 0 and 30 min. Scale bar, 10 µm. Data show the mean ± SEM. * *P* < 0.05; **P < 0.01; ***P < 0.001.

To decipher the molecular mechanism underlying the inhibitory activity of USP25 on microglia activation in vivo and in vitro, western blot analysis was applied to detect the activation of signaling pathways. After ischemic stroke, the NF‐κB and MAPK pathways were activated in the injured hemisphere, and their activity was further enhanced in the absence of USP25 (**Figure** **6**A–E). Consistent with the in vivo observation, USP25 deletion potentiated LPS‐induced activation of NF‐κB and MAPK signaling in cultured BV2 cells (Figure 6F). Upon activation, p65 NF‐κB translocates from cytoplasm to nucleus to mediate gene transcription. Immunofluorescence showed that LPS‐induced nuclear translocation of p65 was strongly increased in USP25‐deficient BV2 cells (Figure 6G), indicating that USP25 inhibits the activation of NF‐κB.

### 2.7 USP25 Inhibits Inflammatory Signal Transduction by Deubiquitinating TAB2

**Figure 7:**
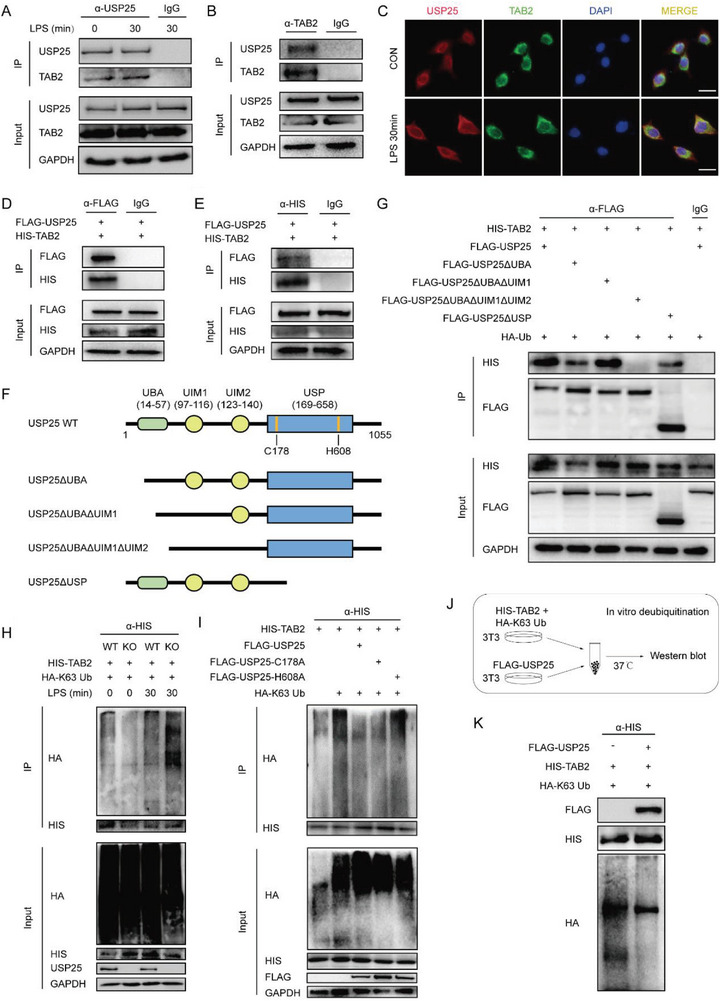
USP25 physically interacts with and K63 deubiquitinates TAB2. A) BV2 cells were left untreated or stimulated with LPS (500 ng ml^−1^) for 30 min. Proteins were immunoprecipitated from whole cell lysates with indicated antibodies and analyzed by western blot. B) Proteins were immunoprecipitated from whole cell lysates of BV2 cells with indicated antibodies and analyzed by western blot. C) Immunofluorescence staining of USP25 (red) and TAB2 (green) in untreated and LPS‐stimulated BV2 cells. Scale bar, 10 µm. D,E) NIH/3T3 cells were transfected with FLAG‐USP25 and HIS‐TAB2 plasmids for 24 h. Proteins were immunoprecipitated from whole cell lysates with indicated antibodies and analyzed by western blot. F) A schematic presentation of USP25 and different mutants. G) USP25^−/−^ NIH/3T3 cells were transfected with indicated plasmids for 24 h. Proteins were immunoprecipitated with indicated antibodies and further analyzed by western blot. H) WT and USP25^−/−^ NIH/3T3 cells were transfected with indicated plasmids for 24 h and then left untreated or treated with LPS (500 ng ml^−1^) for 30 min. Proteins were immunoprecipitated with anti‐HIS antibody and then analyzed by western blot. I) USP25^−/−^ NIH/3T3 cells were transfected with indicated plasmids for 24 h and then stimulated with LPS (500 ng ml^−1^) for 30 min. Proteins were immunoprecipitated with anti‐HIS antibody and analyzed by western blot. J,K) The schematic diagram J) and immunoblots K) of the in vitro deubiquitination assay.

To explore the mechanism by which USP25 regulates NF‐κB and MAPK signaling pathways, we immunoprecipitated USP25 from BV2 cells and analyzed its interaction with common upstream molecules of these signaling pathways. The small‐scale screening identified TAB2 as an interacting protein of USP25 (**Figure** **7**A,B), and USP25 did not interact with other upstream signaling molecules including MyD88, IRAK1, TAK1, and TAB3 (Figure S6, Supporting Information). Besides, LPS stimulation strengthened the interaction between USP25 and TAB2 (Figure 7A). Consistently, immunofluorescence demonstrated that USP25 and TAB2 colocalized in the cytoplasm of BV2 cells and their colocalization was intensified by LPS treatment (Figure 7C). Furthermore, the interaction was consolidated using exogenously expressed FLAG‐tagged USP25 and HIS‐tagged TAB2 (Figure 7D,E). Structurally, USP25 contains a UBA domain, two UIM domains, and a USP domain, which harbors two catalytic residues C178 and H608 (Figure 7F). To determine the domain through which USP25 interacts with TAB2, a series of USP25 mutants lacking certain domains were generated (Figure 7F). As shown in Figure 7G, the USP25 mutant lacking the UIM2 domain failed to interact with TAB2, indicating that USP25 binds to TAB2 through the UIM2 domain. In response to inflammatory stimuli, TAB2 undergoes K63 ubiquitination, which is essential for the activation of TAK1 and downstream signaling molecules.^[^ ^20^ ^]^ We found that USP25 reduced K63‐specific ubiquitination of TAB2 upon LPS stimulation (Figure 7H). Consistently, USP25 ablation increased the K63 polyubiquitination of TAB2 in the brain after MCAO (Figure S7A, Supporting Information). The DUB activity of USP25 is exerted by C178 and H608 residues, and we constructed the FLAG‐USP25‐C178A mutant, whose C178 residue was inactive, and the FLAG‐USP25‐H608A mutant, whose H608 residue was catalytically inactive. Both WT USP25 and the C178A USP25 mutant strongly reduced K63 ubiquitination of TAB2, whereas the H608A USP25 mutant lost the ability to deubiquitinate TAB2 (Figure 7I), showing that the H608 residue is indispensable for the DUB activity of USP25 toward TAB2. In good agreement, USP25 could remove established K63 polyubiquitination chains on TAB2 in an in vitro deubiquitination assay (Figure 7J,K). The K63 polyubiquitination of TAB2 is critical for the recruitment and phosphorylation of TAK1, which then activates downstream NF‐κB and MAPK signaling. Indeed, we found that LPS‐induced interaction between TAB2 and TAK1 was increased in USP25‐deficient BV2 cells (Figure S7B, Supporting Information). Together, these data show that USP25 alleviates LPS‐induced inflammatory signal transduction in microglia by K63 deubiquitinating TAB2.

### 2.8 USP25 Ameliorates Ischemic Stroke Injury by Regulating TAB2

**Figure 8:**
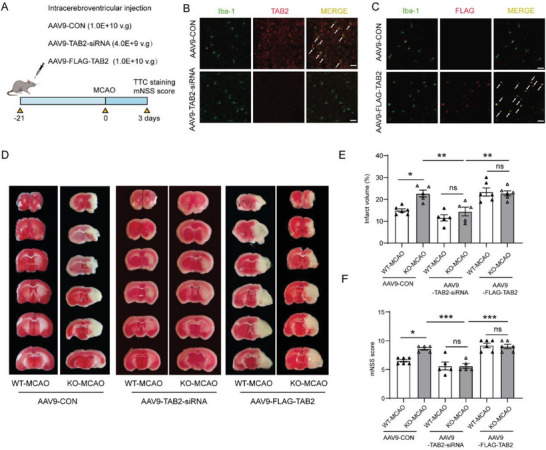
WT and USP25^−/‐^ MCAO mice show comparable disease outcome after TAB2 knockdown or overexpression. A) The experimental flowchart for the MCAO experiment after AAV9 infection. B) Representative immunostaining of Iba‐1 (green) and TAB2 (red) in AAV9‐TAB2‐siRNA‐infected mice on day 21 after injection. Scale bar, 25 µm. C) Representative immunostaining of Iba‐1 (green) and FLAG (red) in AAV9‐FLAG‐TAB2‐infected mice on day 21 after injection. Scale bar, 25 µm. D,E) Representative TTC staining D) and quantification E) of infarct volume in AAV9‐infected WT and USP25^−/−^ mice on day 3 after MCAO. *n* = 5–6 mice per group. ns, not significant. F) mNSS score of AAV9‐infected WT and USP25^−/−^ mice on day 3 after MCAO. *n* = 5–6 mice per group. ns, not significant. Data show the mean ± SEM. * *P* < 0.05; **P < 0.01; ***P < 0.001.

To verify that USP25 ameliorates ischemic stroke injury by regulating TAB2, recombinant adeno‐associated virus serotype 9 (AAV9) vectors carrying TAB2 siRNA or sequence encoding FLAG‐tagged TAB2 were generated and intracerebroventricularly injected into mice to knockdown or overexpress TAB2 in the brain (**Figure** **8**A). Twenty‐one days after virus injection, expression of TAB2 was efficiently altered in brain‐resident cells including microglia (Figure 8B,C; Figure S8, Supporting Information). Knockdown or overexpression of TAB2 blunted the difference in infarct volumes between WT and USP25^−/−^ mice (Figure 8D,E). In addition, Knockdown or overexpression of TAB2 eliminated the difference in mNSS scores between the two genotypes (Figure 8F), demonstrating that USP25 ameliorates MCAO‐induced brain injury by targeting TAB2.

## 3 Discussion

**Figure 9:**
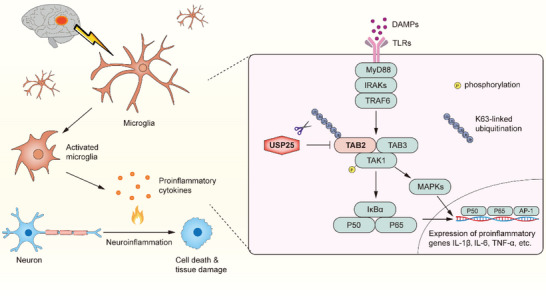
Schematic illustration of the role of USP25 in ischemic stroke injury. After ischemic stroke, microglia are activated and produce proinflammatory cytokines, which promote neuronal death and tissue damage. In this process, USP25 inhibits the activation of NF‐κB and MAPK signaling pathways in microglia by removing K63‐specific polyubiquitin chains from TAB2, thereby ameliorating neuroinflammation and cerebral injury.

Neuroinflammation is a key element underlying the cerebral injury caused by ischemic stroke. In the present study, we demonstrated that ischemic stroke injury was ameliorated by the deubiquitinating enzyme USP25, which reduced neuronal loss and neurological deficits by inhibiting microglia‐mediated inflammatory responses (**Figure** **9**).

USP25 is a DUB that is encoded by a gene located at 21q11.2.^[^ ^21^ ^]^ Functionally, USP25 has been shown to participate in multiple inflammatory diseases including multiple sclerosis,^[^ ^11^ ^]^ sepsis,^[^ ^12^ ^]^ viral infection,^[^ ^13^ ^]^ colitis,^[^ ^15^ ^]^ pancreatitis,^[^ ^14^, ^22^ ^]^ and Alzheimer's disease (AD).^[^ ^16^ ^]^ In addition to inflammatory diseases, USP25 has emerged as a key regulator in cancer.^[^ ^15^, ^23^ ^]^ In the present study, we found that USP25 ameliorated cerebral ischemic stroke injury, expanding the disease spectrum regulated by USP25.

Mechanistically, we found USP25 inhibited TLR4‐induced activation of NF‐κB and MAPK signaling pathways in microglia, which is in line with previous reports that USP25 inhibits these signaling pathways.^[^ ^11^, ^12^ ^]^ Upon stimulation with IL‐17, USP25 cleaves K63‐linked polyubiquitin chains on TRAF5 and TRAF6, leading to the inactivation of these apical signaling molecules and downstream NF‐κB and MAPK pathways.^[^ ^11^ ^]^ Another study by Zhong et al. found that USP25 negatively regulated LPS‐induced NF‐κB and MAPK activation by inhibiting the K48‐specific polyubiquitination and degradation of TRAF3, an inhibitor of TRAF6 in TLR4‐mediated signaling.^[^ ^12^ ^]^ Of note, several DUBs have been shown to regulate a signaling pathway by targeting different signaling molecules. For example, the DUB A20 restricts the activation of NF‐κB by deubiquitinating RIPK1,^[^ ^24^ ^]^ RIPK2,^[^ ^25^ ^]^ TRAF6,^[^ ^26^ ^]^ and NEMO.^[^ ^27^ ^]^ In this study, we identified TAB2 as a new target for USP25. Upon TLR/IL‐1R activation, TAB2 recognizes and binds to K63‐specific polyubiquitin chains on an adaptor, such as TRAF6, through its zinc finger domain, leading to the recruitment of a signaling complex comprising TAK1 and the subsequent activation of the TAK1 kinase.^[^ ^20c^ ^]^ In addition, TAB2 can also trigger the autophosphorylation and activation of TAK1 by binding unanchored K63‐linked polyubiquitin chains.^[^ ^28^ ^]^ TAB3 is a homologue of TAB2 and it can also bind to K63‐linked polyubiquitin chains.^[^ ^20c^ ^]^ However, we found that USP25 specifically interacted with TAB2 but not TAB3. Similarly, a previous report shows that the DUB USP15 regulates TAB2 and TAB3 in different ways.^[^ ^29^ ^]^ Although USP15 K48 deubiquitinates and stabilizes TAB2 through its DUB activity, USP15 inhibits the autophagic degradation of TAB3 independent of its DUB activity.^[^ ^29^ ^]^ Furthermore, we found that USP25 interacted with TAB2 through its UIM2 domain, which is critical for substrate recognition and also required for the interaction between USP25 and TRAF5/6.^[^ ^11^ ^]^ Therefore, USP25 interacted with and K63 deubiquitinated TAB2, thereby inhibiting NF‐κB and MAPK activation in response to LPS.

Noteworthy, TAB2 plays a key role in ischemic stroke injury. A recent study showed that the E3 ligase TRIM45 promoted ischemia‐induced neuronal damage by enhancing microglia‐mediated neuroinflammation.^[^ ^30^ ^]^ Mechanistically, TRIM45 enhances NF‐κB activation by K63 ubiquitinating TAB2.^[^ ^30^ ^]^ In good agreement, we found that USP25 alleviated neuroinflammation and ischemic stroke injury by removing K63 polyubiquitin chains from TAB2. To our knowledge, USP25 is the first DUB that has been found to K63 deubiquitinate TAB2.

In homeostatic brains, USP25 was highly expressed in neurons instead of microglia. Given that neurons expressed high levels of USP25 and a large number of neurons were lost after ischemic stroke, it is not surprising that the overall expression of USP25 was reduced in the brain after MCAO as observed in Figure 1A. However, we found that USP25 deletion or inhibition did not affect the survival of neurons after OGD, indicating that the high abundance of USP25 in neurons is not critically involved in neuronal loss induced by MCAO but may be involved in other pathophysiological conditions rather than ischemic stroke. Indeed, we have found that neuron‐derived USP25 contributes to the pathogenesis and development of AD.^[^ ^16^ ^]^ USP25 promotes the cleavage of amyloid precursor protein (APP) and the generation of amyloid‐β by reducing the ubiquitination and degradation of APP and BACE1, thereby exacerbating AD neuropathology in 5×FAD mice.^[^ ^16^ ^]^

After ischemic insult or inflammatory stimulation, the expression of USP25 was strongly upregulated in microglia. Interestingly, we found that USP25 was also upregulated in peri‐infarct microglia in patients with ischemic stroke. Our experimental data show that USP25 is a key inhibitor of ischemic stroke injury, indicating that therapeutic augmentation of USP25 function might be beneficial for the treatment of ischemic stroke.

## 4 Experimental Section

### Mice

USP25^−/‐^ mice on a C57BL/6 background were kindly provided by Prof. Jian Yuan (Tongji University, China). USP25^−/‐^ mice and wild‐type littermates were kept under SPF conditions in the Laboratory Animal Resources Center of Wenzhou Medical University. Animal care and experimental procedures were performed according to the regulations and approved by the Animal Management and Ethics Committee of Wenzhou Medical University (Approval number: wydw2022‐0740).

### Transient Middle Cerebral Artery Occlusion (MCAO)

Transient MCAO was performed in 10‐12‐week‐old male mice with the intraluminal filament technique.^[^ ^31^ ^]^ During the operation, mice were anesthetized with 2% isoflurane mixed with air, and the body temperature was maintained using a homoeothermic blanket. After the ligation of the common carotid artery and the external carotid, a nylon monofilament with a silicon‐coated tip (RWD life science, Shenzhen, China) was inserted into the left internal carotid artery through a cut in the left external carotid artery. The filament was secured when the silicon‐coated tip obstructed the origin of the middle cerebral artery. Laser Doppler flowmetry (Model P10, Moor Instruments, Wilmington, USA) was applied to monitor the regional cerebral blood flow. The interruption of blood flow in the middle cerebral artery was confirmed when cerebral blood flow dropped to <25% of baseline. After 1 h of obstruction, the filament was withdrawn, and cerebral blood flow returning to >70% of baseline confirmed reperfusion. Sham‐operated mice underwent similar surgical procedures except for filament insertion. After the operation, mice were kept in warm cages for 2 h for recovery. In all MCAO experiments, the examiners were blinded to the group identity of mice.

### 4.1 3, 5‐triphenyltetrazolium Chloride (TTC) Staining

Mice were euthanized and perfused intracardially with saline. After perfusion, the brains were immediately removed and cut into 1‐mm sections in a mold (RWD life science, Shenzhen, China). The sections were incubated in 2% TTC (Solarbio, Beijing, China) staining solution for 15 min at room temperature before fixation with 4% PFA. The images were captured with a digital camera and the infarct volumes were quantified with ImageJ (NIH Image, Bethesda, MD, USA). The infarct percentage of the six sections was calculated after edema correction according to a published method.^[^ ^32^ ^]^

### Neurological Function Assessment

The modified neurological severity score (mNSS), adhesive removal test, corner‐turning test, rotarod test, and handgrip test were performed at 1, 3, 7, and 14 days after reperfusion to assess neurological function. All mice were trained for 3 consecutive days before the MCAO operation. The assessment was carried out by an examiner who was blinded to the group identity of mice.

### Flow Cytometry

Mice were euthanized and the ischemic brain hemispheres were harvested for the generation of single‐cell suspension. Brain‐infiltrating leukocytes and microglia were isolated from the single‐cell suspension by Percoll (Cytiva, UT, USA) gradients as described previously.^[^ ^33^ ^]^ The cells were counted with a hemocytometer. After counting, the cells were stained with fluorochrome‐conjugated antibodies CD45 PerCP (103 129, Biolegend), CD11b FITC (101 205, Biolegend), CD3 BV510 (100 233, Biolegend), CD4 BV421 (100 437, Biolegend), CD8 APC (17‐0088‐41, eBioscience), CD11c PE (117 307, Biolegend), F4/80 BV421 (123 131, Biolegend), Ly‐6C APC (17‐5932‐80, eBioscience), Ly‐6G PE (12‐9668‐82, eBioscience), CD206 APC (17‐2061‐80, eBioscience), CD86 PE (105 105, Biolegend), Rat IgG APC (400 611, Biolegend), and Rat IgG PE (400 607, Biolegend) as indicated. Flow cytometry was performed on a Cytoflex flow cytometer (Beckman Coulter, CA, USA), and data were analyzed with Flowjo software (Informer Technologies, USA).

### Cell Culture and Treatment

The BV2 cell line was purchased from the Cell Resource Center (Shanghai, China) and cultured in DMEM (Gibco, Eggenstein, Germany) supplemented with 10% FBS (ExCell Bio, Shanghai, China) and 1% penicillin/streptomycin (Solarbio, Beijing, China). Primary astrocytes were isolated from newborn mice^[^ ^34^ ^]^ and cultured in DMEM supplemented with 10% FBS and 1% penicillin/streptomycin. Next, mixed glial cells were cultured for 14–15 days until a confluent monolayer to isolate primary microglia.^[^ ^31^ ^]^ Then, DMEM medium mixed with 0.25% trypsin (2:1) was added to remove astrocytes. The remaining adherent cells were primary microglia of high purity and were used for further analysis. The SH‐SY5Y cell line was purchased from the Cell Resource Center (Shanghai, China) and cultured in DMEM/F‐12 (Gibco, Eggenstein, Germany) supplemented with 10% FBS and 1% penicillin/streptomycin. NIH/3T3 cell line was purchased from Shanghai Institute of Biochemistry and Cell Biology (Shanghai, China) and cultured in DMEM supplemented with 10% FBS and 1% penicillin/streptomycin. NIH/3T3 cells were transfected with indicated plasmids (GeneChem, Shanghai, China) with Lipofectamine^TM^ 3000 Transfection Reagent (Thermo Fisher Scientific, MA, USA) according to the manufacturer's instructions. For studying signal transduction and cytokine production, cells were treated with 500 ng ml^−1^ LPS (Solarbio, Beijing, China). For USP25 inhibition, cells were treated with 5 µm AZ1 (MedChemExpress, NJ, USA).

### Generation of USP25 Knockout Cell Lines

USP25 gRNA (AGTGCACACAGGTTTACTGG) was inserted into the lentiCas9‐Blast plasmid (#52 962, Addgene). The lentiCas9‐Blast‐USP25 gRNA plasmids were then co‐transfected into 293T cells together with pMD2.G (#12 259, Addgene) and psPAX2 (#12 260, Addgene) plasmids for virus production, and lentivirus‐containing supernatant was collected 48 h after transfection. BV2 cells were transduced with the lentivirus in the presence of 6 µg ml^−1^ polybrene (Sigma Aldrich). On day 3 after transduction, 4 µg ml^−1^ blasticidin (Yeasen Biotechnology, Shanghai, China) was added to select single‐cell clones. The USP25^−/−^ NIH/3T3 cells were generated similarly. The deletion of USP25 in selected cell clones was verified by western blot.

### Oxygen‐Glucose Deprivation (OGD)

OGD was performed to mimic the ischemic attack in vitro as published.^[^ ^35^ ^]^ Briefly, cells cultured in medium deprived of serum and glucose were placed in a hypoxic chamber with 95% N_2_ and 5% CO_2_. The chamber was then incubated at 37 °C for the indicated time periods. After OGD, cells were removed from the hypoxic chamber and incubated in glucose‐containing medium in an incubator with 95% air and 5% CO_2_ for the indicated time periods.

### Quantitative Real‐Time qPCR

Total mRNA was isolated from cells and brain tissue with TRIzol (Invitrogen, CA, USA) regent, and cDNA was subsequently synthesized using the PrimeScript™ RT reagent kit (Takara, Kyoto, Japan). Quantitative Real‐time PCR for *Usp25*, *Il6*, *Il1b*, *Tnfα*, *Cxcl10, Ccl2*, *S100a8*, *S100a9* and *Actb* was performed with ChamQ Universal SYBR qPCR Master Mix (Vazyme, Nanjing, China) on a QuantStudio 3^TM^ Real‐Time PCR System (Thermo Fisher Scientific, MA, USA). All primers were purchased from Sangon Biotech (Shanghai, China) and primer sequences are included in Table S1 (Supporting Information). Data were analyzed using the 2^−ΔΔCt^ algorithm, and the results were displayed as fold increase over naive controls.

### Western Blot

Brain tissue and cells were lysed in RIPA lysis buffer (Beyotime Biotechnology, Shanghai, China) supplemented with protease inhibitor cocktail and PMSF (Solarbio, Beijing, China). Cell lysates were centrifuged at 14 000 g at 4 °C for 10 min to pellet debris. The supernatant was harvested and protein concentration was determined using the Quick Start Bradford 1x Dye Reagent (BIO‐RAD, CA, USA). Equal amounts of samples were separated by SDS‐PAGE and then transferred to PVDF membranes. After blocking with 5% BSA, membranes were probed for USP25 (Abcam, ab187156, 1:1000), PSD95 (Proteintech, 20665‐1‐AP, 1:1000), NeuN (Abcam, ab177487, 1:1000), TAB2 (Proteintech, 14410‐1‐AP, 1:1000), TAB3 (Abcam, ab124723, 1:1000), MyD88 (Cell Signaling Technology, 4283S, 1:1000), IRAK1 (Proteintech, 10478‐2‐AP, 1:1000), TAK1 (Cell Signaling Technology, 5206S, 1:1000), phospho‐p65 (Cell Signaling Technology, 3033S, 1:1000), p65 (Cell Signaling Technology, 8242S, 1:1000), phospho‐ERK1/2 (Cell Signaling Technology, 4370S, 1:1000), ERK1/2 (Cell Signaling Technology, 4695S, 1:1000), phospho‐p38 (Cell Signaling Technology, 9211S, 1:1000), p38 (Cell Signaling Technology, 9212S, 1:1000), phospho‐JNK (Cell Signaling Technology, 4668S, 1:1000), JNK (Cell Signaling Technology, 9252S, 1:1000), IκBα (Cell Signaling Technology, 4814S, 1:1000), Cleaved Caspase‐3 (Cell Signaling Technology, 9664S, 1:1000), Caspase‐3 (Affinity, AF6311, 1:1000), His‐Tag (Proteintech, 66005‐1‐Ig, 1:1000), FLAG tag (Proteintech, 66008‐4‐Ig, 1:1000), HA‐Tag (Proteintech, 51064‐2‐AP, 1:1000), and GAPDH (Bioworld, MB001, 1:10 000). After overnight incubation with primary antibodies at 4 °C, membranes were incubated with corresponding secondary antibodies at room temperature for 1 h. WB images were developed with the ECL Plus Kit (GE Healthcare) and captured on a ChemiDoc XRS+ Gel Imaging System (Bio‐Rad, CA, USA). Uncropped original blots were included in the supplementary material.

### Immunoprecipitation

Protein samples were generated from cells or brain tissue as described in “Western Blot”. Samples were incubated with sepharose beads (Beyotime Biotechnology) under gentle rotation at 4 °C for 2 h to remove unspecific bead‐binding proteins. After that, samples were centrifuged at 12 000 g at 4 °C for 5 min to pellet the pre‐clearing beads. The supernatant was harvested and equal amounts of protein samples were incubated with indicated antibodies at 4 °C overnight. Immunocomplexes were captured by incubating with sepharose beads under gentle rotation at 4 °C for 2 h. The beads were washed 5 times with ice‐cold PBS before further analysis.

### In Vitro Deubiquitination Assay

NIH/3T3 cells were transfected with FLAG‐USP25 plasmids or HIS‐TAB2 + HA‐K63 Ub plasmids for 24 h. After treatment with 500 ng ml^−1^ LPS for 30 min, cells were lysed with RIPA buffer and K63 ubiquitinated TAB2 was harvested from the cell lysate by immunoprecipitation with anti‐HIS antibody. FLAG‐USP25 was harvested by immunoprecipitation with anti‐FLAG antibody. After washing with PBS, the immunoprecipitated samples were washed with deubiquitination buffer (50 mm Tris‐HCl, 5 mm MgCl_2_, 2 mm DTT, 2 mm ATP‐Na_2_, 5% glycerol). Then, K63 ubiquitinated TAB2 was incubated with or without FLAG‐USP25 in the deubiquitination buffer at 37 °C for 2 h. After incubation, western blot was applied to detect the K63 ubiquitination of TAB2.

### Clinical Samples

Cortical and subcortical biopsies were obtained from non‐eloquent brain areas prior to tumor removal (cerebral metastases or high‐grade gliomas) from participants undergoing scheduled tumor surgery at the Department of Neurosurgery, Odense University Hospital. All participants gave their informed written consent to participate in the study. Only participants that had not received prior radiotherapy were included. The samples were stored in iced, sterile Ringer Lactate until further processing.

*Postmortem* brain tissue from four stroke cases was obtained from the Department of Pathology, Odense University. Parallel tissue sections were used in previous studies.^[^ ^7^, ^36^ ^]^ The use of human material was approved by the Regional Ethical Committee in the Region of Southern Denmark (S‐20130048, S‐1300854, and S‐20220018) and was performed in agreement with the declaration of Helsinki. The study was registered with the Danish Data Protection Agency.

### Immunofluorescence

For mouse tissue staining, mice were euthanized and perfused with normal saline, followed by perfusion with 4% PFA. Mouse brains were isolated and fixed in 4% PFA for 48 h before embedding in paraffin. Then, brains were cut into 5 or 20 µm slices and placed on glass slides. After blocking with 5% BSA for 30 min at 37 °C, brain sections were stained with primary antibodies against USP25 (Santa Cruz Biotechnology, sc‐398414, 1:50), NeuN (Abcam, ab177487, 1:200), GFAP (Santa Cruz Biotechnology, sc‐33673), Iba‐1 (Cell Signaling Technology, 17198S, 1:200), TNF‐α (Proteintech, 60291‐1‐Ig, 1:50), and TAB2 (Proteintech, 14410‐1‐AP, 1:200) overnight at 4 °C. Thereafter, brain sections were incubated with fluorescence‐conjugated secondary antibodies (Yeasen Biotechnology) at 37 °C for 1 h. For cell staining, cells were fixed in 4% PFA for 15 min and then incubated in 0.5% Triton X‐100 for 20 min. After blocking with 5% BSA at 37 °C for 30 min, cells were stained with primary antibodies against p65 (Cell Signaling Technology, 8242S, 1:200), USP25 (Santa Cruz Biotechnology, sc‐398414, 1:50), and TAB2 (Proteintech, 14410‐1‐AP, 1:200). Then, cells were incubated with fluorescence‐conjugated secondary antibodies (Yeasen Biotechnology) at 37 °C for 1 h. DAPI was used to stain the nucleus. Images were captured with a Nikon microscope imaging system (Nikon, Japan) and analyzed with ImageJ (NIH Image, Bethesda, MD, USA).

Human biopsies were immersion fixated in 4% PFA, cryopreserved in 20% sucrose, frozen in gaseous CO_2_, cut into 20 µm‐thick sections on a cryostat, and placed on gelatin‐coated glass slides. *Postmortem* brain tissue from ischemic stroke cases was formalin‐fixed, embedded in paraffin, and cut into 2 µm‐thick sections on a microtome. Tissue sections were deparaffinized in xylene and rehydrated in graded series of ethanol (99%, 96%, 70%, 50%), emerged in water, and washed in PBS before heat‐induced epitope retrieval in citrate buffer (10 mm citrate, pH 6). Next, sections were rinsed in PBS and bleached using the Autofluorescence Eliminator Reagent (Millipore, Soeborg, Denmark) according to the manufacturer's guidelines. The sections were then rinsed in PBS followed by TBS and TBS + 0.1% triton before blocking in 10% FCS in TBS for 30 min at room temperature. The sections were incubated overnight with primary antibodies directed against USP25 (1:50) and Iba1 (Fujifilm, 019–19741, 1:200) diluted in 10% FCS in TBS. The following day, the sections were rinsed in TBS + 0.1% triton, and incubated for 2 h with secondary antibodies (1:200, Invitrogen) diluted in 10% FCS in TBS at room temperature, protected from light. Finally, sections were rinsed in TBS before mounting with ProLong Gold Antifade Reagent with DAPI. Images were captured on an Olympus BX53 microscope.

### TUNEL Staining

After washing twice with PBS, cells were fixed in 4% PFA for 30 min at 4 °C. Then, cells were permeabilized with PBS containing 0.2% Triton X‐100 for 20 min at room temperature. After washing twice with PBS, cells were incubated with 50 µl of TUNEL Reaction Buffer containing 1 µl TdT enzyme (Proteintech, PF00006) for 60 min at 37 °C. Images were captured on a Zeiss microscope imaging system (Zeiss, LSM 980 with Airyscan2, Germany) and analyzed by ImageJ.

### AAV9‐Mediated RNAi

Mice were intracerebroventricularly injected with adeno‐associated virus serotype 9 (AAV‐9) carrying TAB2 siRNA or sequence encoding FLAG‐tagged TAB2 (GeneChem, Shanghai, China), which transduced brain‐resident cells including microglia. The injection coordinates from bregma were −0.22 mm anterior, +1.00 mm left lateral, and −2.20 mm ventral. In total, AAV9‐CON (1.0E+13 v.g), AAV9‐TAB2‐RNAi (4.0E+12 v.g), or AAV9‐FLAG‐TAB2 (1.0E+13 v.g) in 1 µl volume was injected into each mouse at the speed of 200 nl min^−1^. After injection, the mice were rested for 3 weeks before further analysis.

### Statistics

Statistical analysis was performed with GraphPad Prism 7.0 software (GraphPad, San Diego, USA). Two‐tailed Student's *t* test was applied to detect differences between two groups and one‐way ANOVA was used for multiple comparisons. *P* values < 0.05 were considered to be statistically significant.

## Conflict of Interest

The authors have no conflict of interest.

## Supporting information

Supporting Information

## Data Availability

The data that support the findings of this study are available from the corresponding author upon reasonable request.
