# Supplementary material for: USP25 Inhibits Neuroinflammatory Responses After Cerebral Ischemic Stroke by Deubiquitinating TAB2
Source: Adv Sci (Weinh). 2023 Aug 16;10(28):2301641. doi: 10.1002/advs.202301641 (PMC10558664; doi:10.1002/advs.202301641)
Supplement: Supplementary file 1 — Supporting Information [file ADVS-10-2301641-s001.pdf]

ADVANCED SCIENCE
Open Access

Supporting Information

for *Adv. Sci.*, DOI 10.1002/advs.202301641

USP25 Inhibits Neuroinflammatory Responses After Cerebral Ischemic Stroke by Deubiquitinating TAB2

Zhongding Li, Baohua Liu, Kate Lykke Lambertsen, Bettina Hjelm Clausen, Zhenhu Zhu, Xue Du, Yanqi Xu, Frantz Rom Poulsen, Bo Halle, Christian Bonde, Meng Chen, Xue Wang, Dirk Schlüter, Jingyong Huang, Ari Waisman, Weihong Song and Xu Wang**

Supporting Information

USP25 inhibits neuroinflammatory responses after cerebral ischemic stroke by deubiquitinating TAB2

Zhongding Li[#], Baohua Liu[#], Kate Lykke Lambertsen, Bettina Hjelm Clausen, Zhenhu Zhu, Xue Du, Yanqi Xu, Frantz Rom Poulsen, Bo Halle, Christian Bonde, Meng Chen, Xue Wang, Dirk Schlüter, Jingyong Huang, Ari Waisman, Weihong Song[*], Xu Wang[*]

Supplementary Table S1: qPCR primer sequences

Gene	Species	Sequence
Actb	Mouse	CTACCTCATGAAGATCCTGACC
		CACAGCTTCTCTTTGATGTCAC
Il1b	Mouse	TCGCAGCAGCACATCAACAAGAG
		AGGTCCACGGGAAAGACACAGG
Tnfα	Mouse	ATGTCTCAGCCTCTTCTCATTC
		GCTTGTCACTCGAATTTTGAGA
Il6	Mouse	CTCCCAACAGACCTGTCTATAC
		CCATTGCACAACTCTTTTCTCA
Ccl2	Mouse	TTAAAAACCTGGATCGGAACCAA
		GCATTAGCTTCAGATTTACGGGT
Cxcl10	Mouse	CAACTGCATCCATATCGATGAC
		GATTCCGGATTCAGACATCTCT
Usp25	Mouse	AATGTTCGGTCAGTATCCACTT
		ATCGGAGTGTAAGGATTCGATC
Actb	Human	CCTGGCACCCAGCACAAT
		GGGCCGGACTCGTCATAC
S100a8	Human	ATGCCGTCTACAGGGATGAC
		ACTGAGGACACTCGGTCTCTA
S100a9	Human	GGTCATAGAACACATCATGGAGG
		GGCCTGGCTTATGGTGGTG

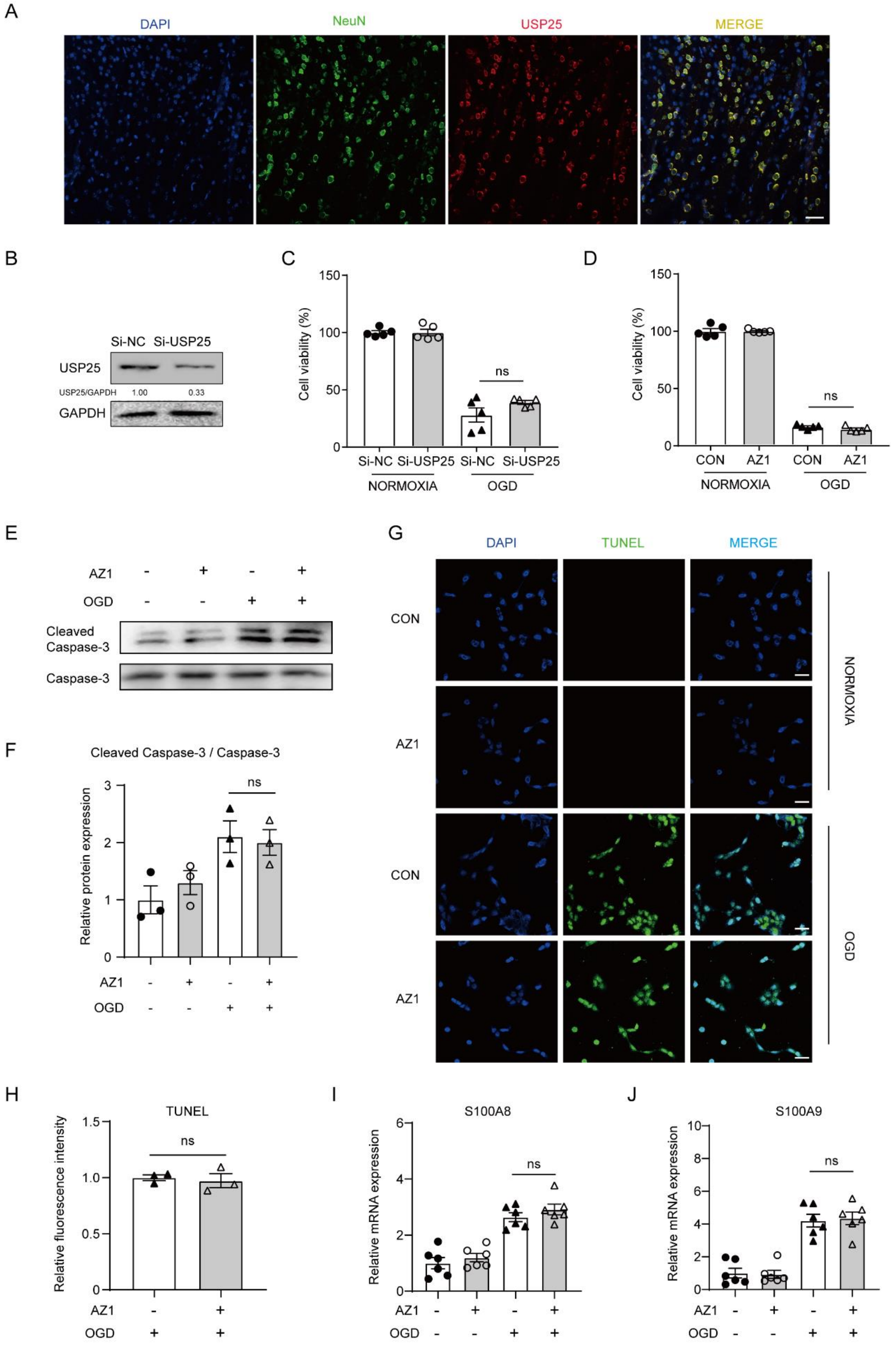

Supplementary Figure 1. USP25 does not affect OGD-induce neuronal death and

DAMP production

(A) Representative immunostaining of NeuN (green) and USP25 (red) in normal brain tissue of C57BL/6 mice. Scale bar, 100 μm.

(B) SH-SY5Y cells were transfected with nonsense siRNA (Si-NC) or USP25 siRNA (Si-USP25) for 48 h. Western blot was performed to detect USP25 levels.

(C-D) After siRNA transfection (C) or AZ1 treatment (D), SH-SY5Y cells were left untreated or subjected to OGD for 6 h followed by reoxygenation for 18 h. CCK8 assay was applied to detect cell viability.

(E-F) After AZ1 treatment, SH-SY5Y cells were left untreated or subjected to OGD for 6 h followed by reoxygenation for 18 h. Representative result (E) and quantification (F) of Cleaved Caspase-3 immunoblotting.

(G-H) After AZ1 treatment, SH-SY5Y cells were left untreated or subjected to OGD for 6 h followed by reoxygenation for 18 h. Representative fluorescence image (G) and quantification (H) of TUNEL staining. Scale bar, 50 μm.

(I-J) After AZ1 treatment, SH-SY5Y cells were left untreated or subjected to OGD for 6 h followed by reoxygenation for 18 h. The transcription of S100A8 (I) and S100A9 (J) was analyzed by qRT-PCR.

Data show the mean ± SEM. ns, not significant.

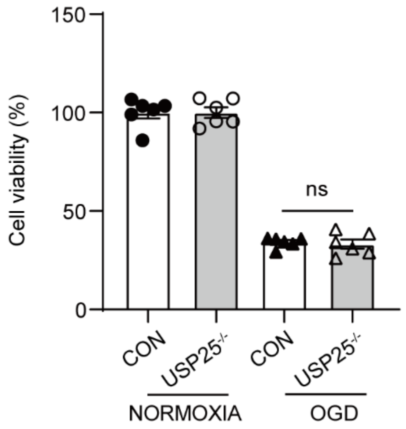

Supplementary Figure 2. USP25 does not affect OGD-induce death of microglia

BV2 cells were left untreated or subjected to OGD for 6 h followed by reoxygenation for 18 h. CCK8 assay was applied to detect cell viability.

Data show the mean ± SEM. ns, not significant.

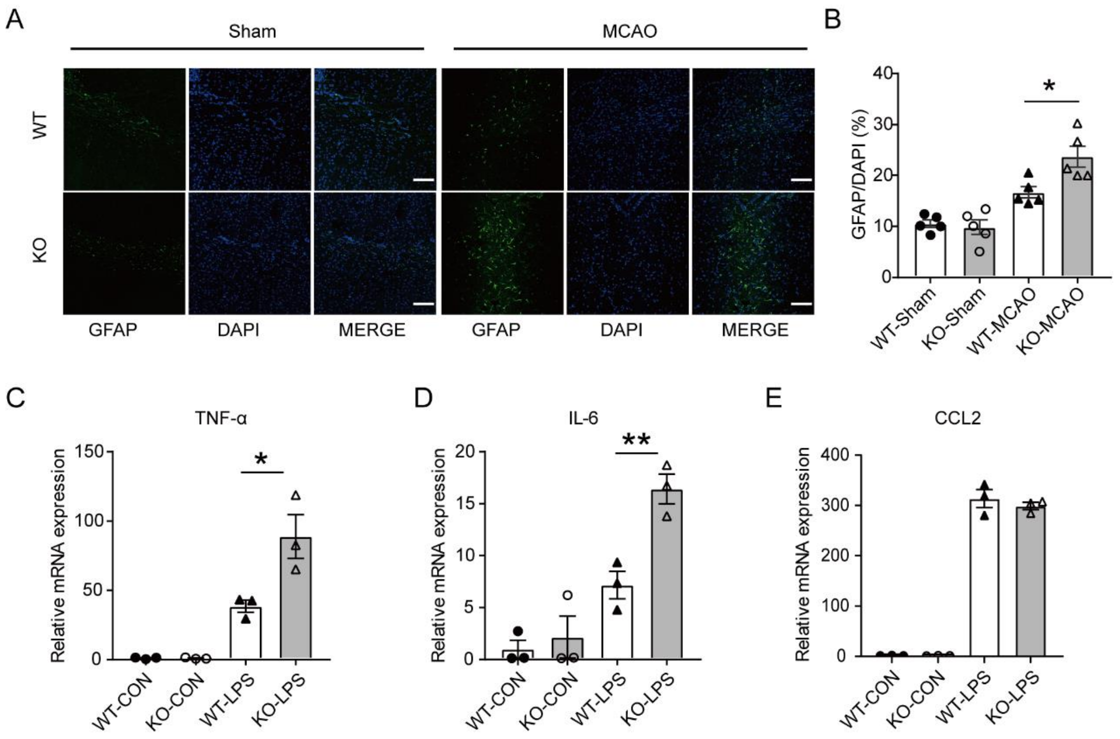

Supplementary Figure 3. USP25 deficiency increases the number and activation of astrocytes

(A-B) Representative immunofluorescence staining (A) and percentages (B) of $GFAP^+$ cells in the ischemic penumbra on day 2 after MCAO. Scale bar, 100 µm, n = 5 mice per group.

(C-E) Transcription of TNF-α (C), IL-6 (D), and CCL2 (E) in control and $USP25^{-/-}$ primary astrocytes was detected by qRT-PCR after stimulation with LPS (500 ng/ml) for 16 h. n = 3 per group.

Data represent the mean ± SEM. * $P < 0.05$; **$P < 0.01$; ***$P < 0.001$.

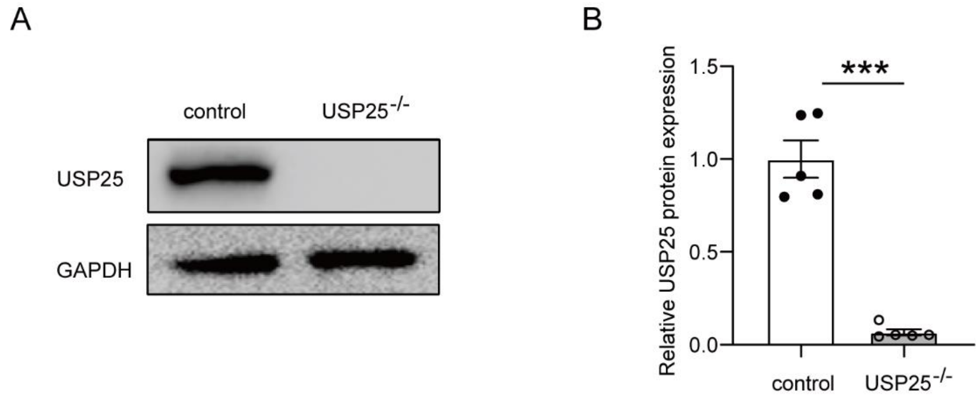

Supplementary Figure 4. USP25 is efficiently deleted in $USP25^{-/-}$ BV2 cells

(A-B) Representative immunoblotting (A) and quantification (B) of USP25 expression in WT and $USP25^{-/-}$ BV2 cells.

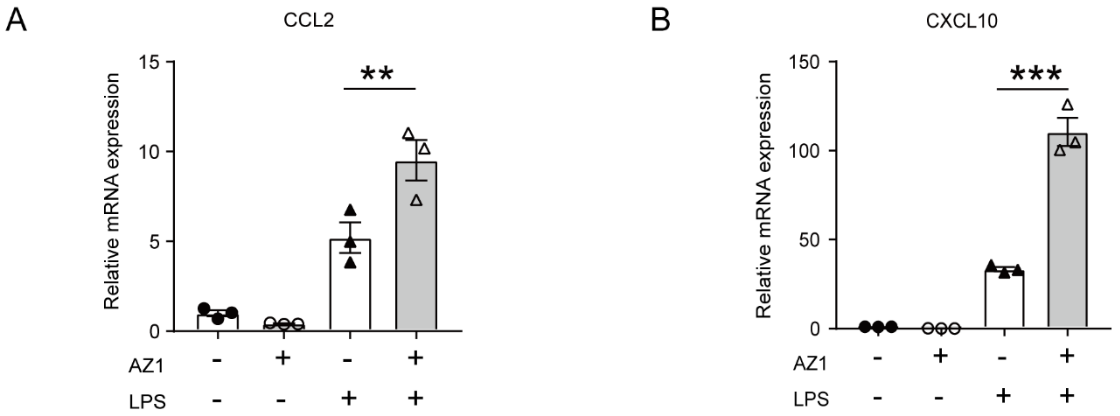

Supplementary Figure 5. AZ1 treatment increases LPS-induced chemokine production in BV2 cells

(A to B) BV2 cells were treated with AZ1 (5 μM) for 2 h, followed by stimulation with LPS (500 ng/ml) for 3 h. The transcription of CCL2 (A) and CXCL10 (B) was detected by qRT-PCR.

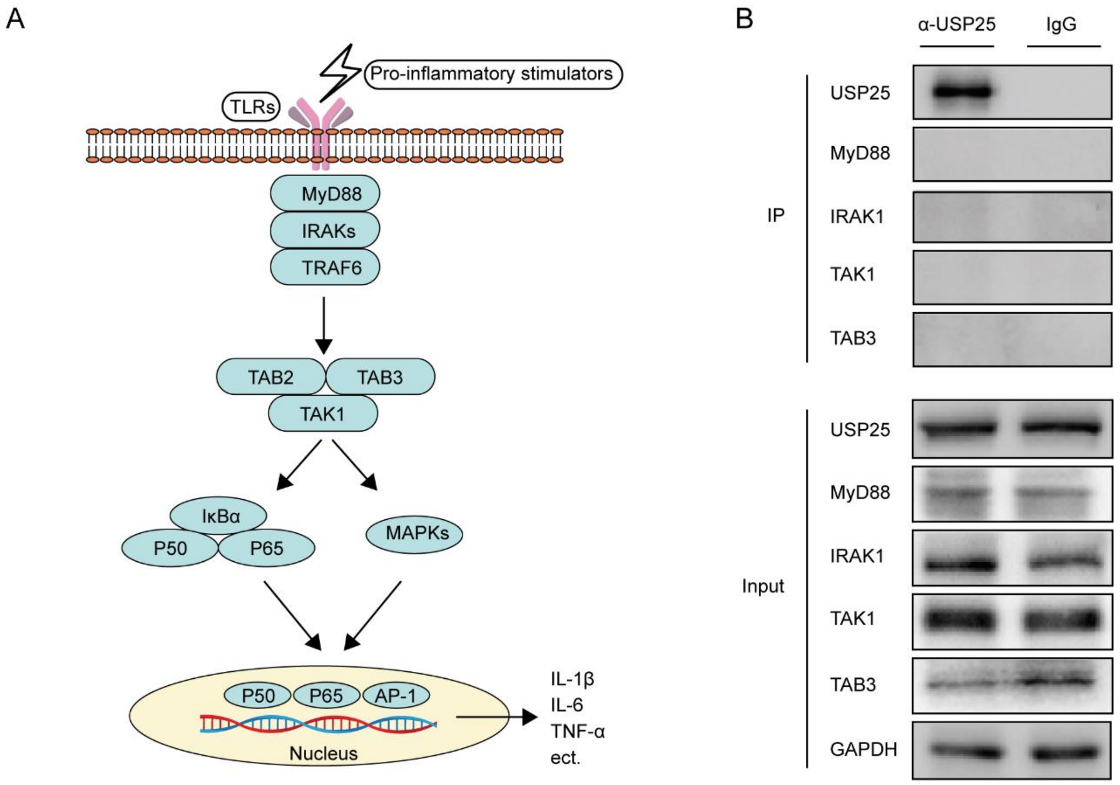

Supplementary Figure 6. Screening for USP25-interacting signaling molecules

(A) Schematic illustration of TLR4-mediated signaling transduction.

(B) Proteins were immunoprecipitated from BV2 whole cell lysates with anti-USP25 antibody or IgG, and subsequently analyzed by western blot with indicated antibodies.

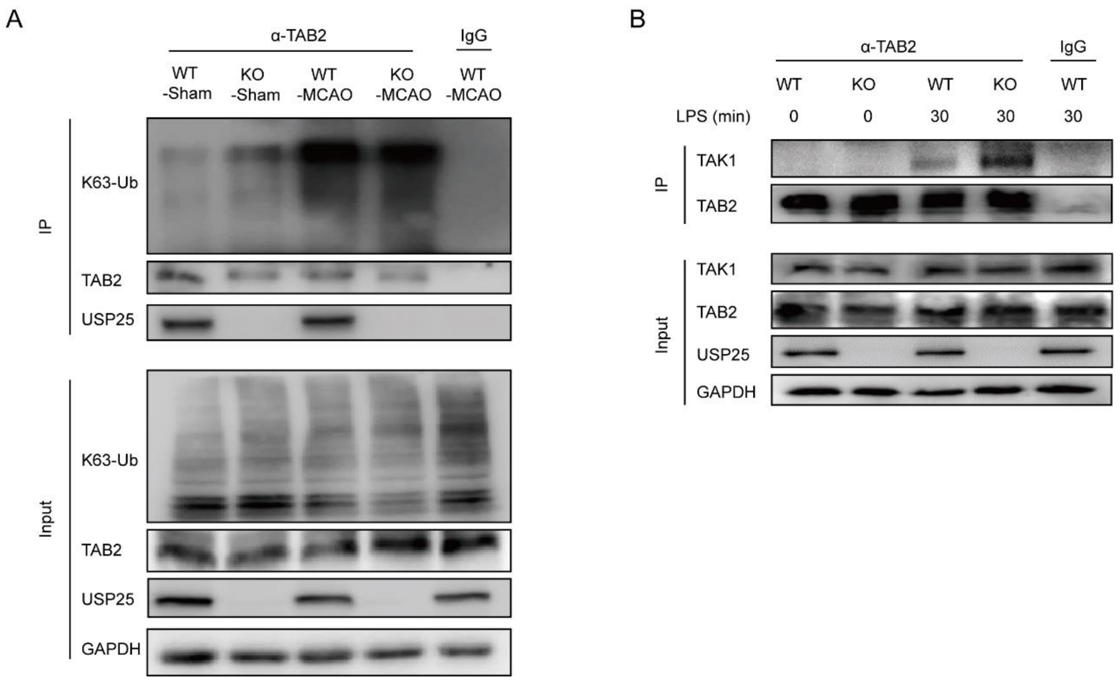

Supplementary Figure 7. USP25 inhibits K63 ubiquitination of TAB2 and TAB2-TAK1 interaction

(A) The ischemic brain hemisphere was harvested on day 2 after MCAO and lysed for protein isolation. Proteins were immunoprecipitated with anti-TAB2 antibody and then analyzed by western blot with indicated antibodies. n = 4 mice per group.

(B) WT and USP25$^{-/-}$ BV2 cells were left untreated or treated with LPS (500 ng/ml) for 30 min. Proteins were immunoprecipitated with anti-TAB2 antibody and then analyzed by western blot.

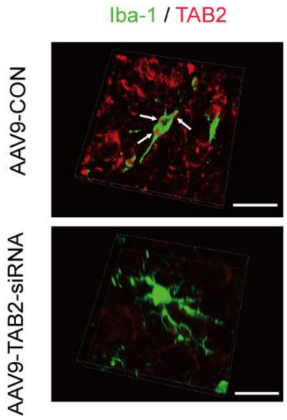

Supplementary Figure 8. TAB2 is silenced in microglia after AAV9-TAB2-RNAi treatment

Z-stack confocal images of Iba-1 (green) and TAB2 (red) in AAV9-TAB2-RNAi infected mice on day 21 after injection. Scale bar, 20 μm.
